# Trends in Traumatic Brain Injuries During the COVID-19 Pandemic: A Single-Center Review of Patient Charts From Pakistan

**DOI:** 10.7759/cureus.58745

**Published:** 2024-04-22

**Authors:** Salaar Ahmed, Ammar Anwer, Muhammad Abdullah, Mohammad Ashraf, Javed Iqbal, Javaria Siddiq, Naveed Ahmed Khan, Hira Khan

**Affiliations:** 1 Medical College, Aga Khan University, Karachi, PAK; 2 Department of Neurosurgery, University College of Medicine and Dentistry, The University of Lahore, Lahore, PAK; 3 Department of Neurological Surgery, Wolfson School of Medicine, University of Glasgow, Glasgow, GBR; 4 Department of Neurosurgery, Mayo Hospital, Lahore, PAK; 5 Department of Plastic Surgery, University College of Medicine and Dentistry, The University of Lahore, Lahore, PAK

**Keywords:** pakistan, low middle-income countries, neurotrauma, road traffic accidents, traumatic brain injury, covid 19

## Abstract

Introduction

A traumatic brain injury (TBI) is one of the leading causes of injury-related deaths, making it a public health concern of extreme importance. In a developing country such as Pakistan, TBIs are significantly underreported, with the treatment frequently being delayed and inadequate, especially in rural healthcare setups all across the country. This concern is further magnified by insufficient epidemiological data on TBIs available in Pakistan. The coronavirus disease 2019 (COVID-19) pandemic brought consequential changes to the healthcare system with the priority shifting toward COVID-19 patients, resulting in considerable changes to the workflow and management of TBIs. The primary objective of this study is to offer valuable insights into the epidemiology of TBIs in Pakistan and its relationship with the impact of the COVID-19 pandemic.

Methods

A retrospective study was conducted at a tertiary care center in a metropolitan city in Pakistan. Patient charts were reviewed from January to August 2020, and data was extracted including demographics, clinical presentation, management, and outcomes for cases of TBI.

Results

The total number of patients is 2126, male 78% and female 21.4%. The mean age of the patients was 28.85. The state of admissions at the hospital is at 99.7% for EME admissions and 0.282% for OPD admissions. Participants presented with loss of consciousness (70.7%), nosebleeds, (53.2%), vomiting (69.0%), and seizures (11.5%). The majority (51.1%) were related to road traffic accidents, followed by falls (20.7%), and assaults (4%). While 1202 (58.5%) of these were managed conservatively, others underwent surgical treatment in the form of craniotomy (28.0%), Burr holes (3.20%), and fracture elevation and repair (10.5%). A decrease in the number of reported TBI cases was observed with lockdown implementation in Pakistan.

Conclusion

The transportation sector in Pakistan was severely affected by the COVID-19 pandemic, leading to a decline in road traffic injuries and TBIs. Stringent mobility constraints and changes in societal and cultural norms have contributed to this reduction.

## Introduction

Traumatic brain injuries (TBIs) are a public health concern of grave importance worldwide, with over one-third of all injury-related deaths being attributed to them. This predicament causes neurological disability, leaving victims unable to lead regular lives. Road traffic accidents (RTAs) are one of the primary causes of TBIs, resulting in an alarming rise in deaths and injuries globally. Reports reveal that over 1.35 million people die annually from RTAs, while 20-50 million people suffer non-fatal wounds. Low and low-middle-income countries like Pakistan suffer the most, experiencing 93% of fatalities resulting from these accidents [1-4].

Head trauma now often termed as TBI carries the worst prognosis and yields significant mortality in patients when compared to patients suffering any other traumatic injury [5]. Unfortunately, in Pakistan, TBIs are not only significantly underreported but also misdiagnosed, with treatment often delayed and/or inadequate, especially in non-urban healthcare setups all over the country [6-8]. This issue is further compounded by the lack of information, with little epidemiological data available on this issue, and the available data mainly encompassing larger urban areas. This drawback is cause for concern, as the majority of Pakistan's citizens reside in rural areas where open vehicles such as motorcycles are prevalent and law enforcement is lacking [9].

Despite the urgency of the situation, Pakistan's public sector-based healthcare system is grappling with implementing a uniform policy or standardized protocol for identifying and managing trauma in the aftermath of an RTA. This inconsistency is prevalent among hospitals and physicians, causing significant delays in necessary treatments. The burden of TBIs resulting from RTAs not only detrimentally affects individuals and their families, but also incurs substantial economic costs for Pakistan's healthcare system [10].

On February 26th, 2020, Pakistan reported its first case of COVID-19, which was followed by varying degrees of lockdown throughout the year. The COVID-19 pandemic resulted in a high influx of patients, particularly those with severe infection, in healthcare facilities. To accommodate this, healthcare services had to reallocate their resources and prioritize COVID-19 patients. As a result, there were several changes to the workflow and management of TBIs, especially since these too were acute cases requiring urgent care like COVID-19. Additionally, measures aimed at reducing the transmission of the infection, such as social distancing and enhanced sanitation protocols, have also affected TBI management [11].

The purpose of this study is to analyze the retrospective data from this time period and answer important questions about how the pandemic impacted TBIs in Pakistan. We have used the COVID-19 pandemic as an external shock to study its impact on the epidemiology of TBIs, as reported by patient charts at a tertiary care hospital in Lahore. Additionally, we will explore the primary reasons for any changes or patterns noted in TBIs.

## Materials and methods

A retrospective study was conducted at the Department of Neurosurgery, Mayo Hospital, a tertiary care center in Lahore, Pakistan, and one of the five major designated government-funded hospitals for head injury. Patient records from hospital admission charts were retrieved for all patients who presented with TBIs between January 2020 and August 2020. Since the study involved a retrospective analysis of data collected as part of routine patient care, an exemption was obtained from the surgical department’s ethical review board. Primary consent was obtained at the time of admission from either the patient or their attendant, which included consent for data usage for research purposes. 

Immediate cases of death (within 24 hours) or patients with minor injuries discharged without the need for admission were not included as part of the study. Pediatric trauma patients were included in the study since no definite age group was defined. Patients with incomplete or incorrect demographic records, as retrieved in the hospital record were excluded from the study. Data analysis was conducted using R, version 4.2.0 (R statistical software; R Foundation for Statistical Computing, Vienna, Austria). Categorical variables such as the gender of the patient, type of injury, and management were reported as numbers and frequencies, whereas continuous variables such as age were presented as means and standard deviations. Specific quantitative variables, such as the Glasgow Coma Scale (GCS) used for assessing the severity of injury and the Glasgow Outcome Scale (GOS) used for assessing outcome and recovery, were calculated and further dichotomized. 

## Results

A total of 2126 cases of TBIs were retrieved using the record. The vast majority were men (n=1659, 78%), with less than a quarter of those injured being women (n=454, 21.4%). The mean age of the study cohort was 28.85 years (SD: 19.65). Further information on the demographics of patients is provided in Table 1. Table 1 also presents the etiology of injury, the major presenting complaints, and the post-resuscitation GCS in the study cohort. Road traffic accidents were the most prevalent cause of head injury (n=1086, 51.1%) and loss of consciousness was the most prevalent presenting complaint (n=1503, 70.7%). Head injury classified by the Glasgow Coma Scale Score (GCS) was mild in most patients (n=786, 37%).

**Table 1:** Demographic and presenting status of patients admitted for traumatic brain injury (TBI) GCS: Glasgow Coma Scale

Gender	Frequency (N= 2126) (%)	Mean Age (Years)
Male	1659 (78%)	28.6
Female	454 (21.4%)	29.7
Not specified	13 (0.6%)	24
Type of Injury	Absolute Numbers	Percentage (%)
Road Traffic Accident	1086	51.1%
Fall	440	20.7%
Assault	85	4%
Unspecified / Others	515	24.2%
Presenting Complaint		
Loss of Consciousness	1503	70.7%
Nosebleed	1130	53%
Vomiting	1468	69%
Seizures	245	11.5%
GCS Post-Resuscitation		
Mild	786	37%
Moderate	626	29.4%
Severe	426	20%
Not available	288	13.5%

**Figure 1:**
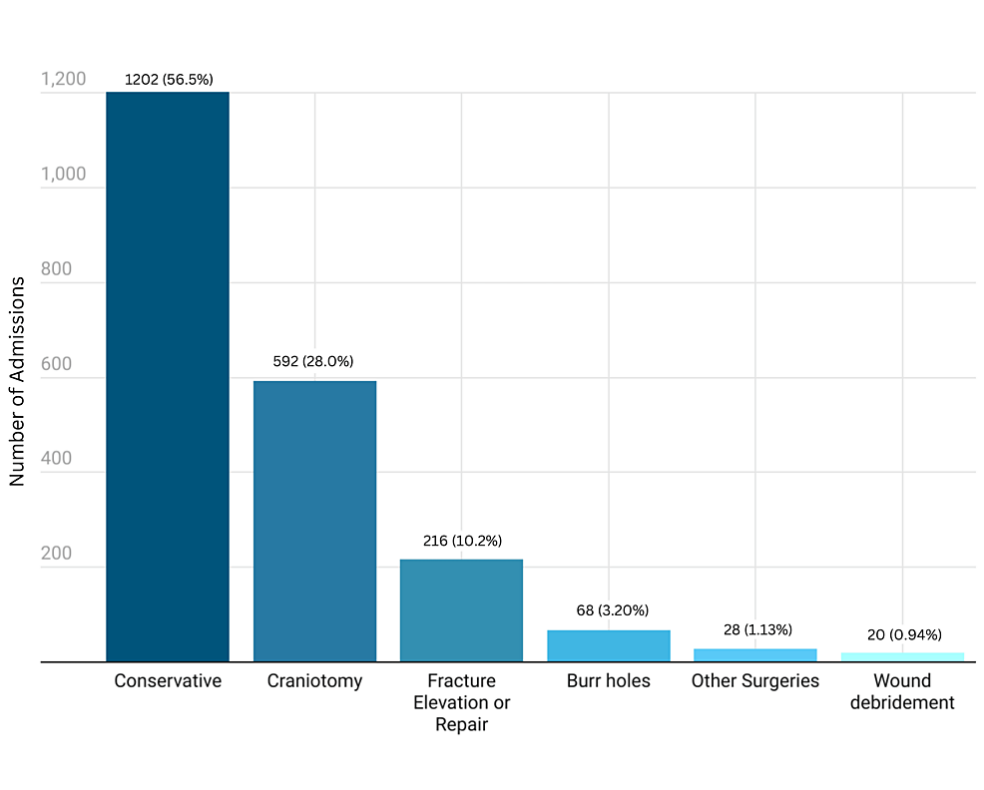
Management of traumatic brain injury (TBI) cases admitted at a tertiary care hospital in Lahore between January and August 2020

Whilst the majority of patients were managed conservatively, with observation and appropriate medical treatment (n=1202, 56.5%), the remaining nearly half required some form of surgical intervention with a craniotomy being the most performed procedure (n=595, 28%). Figure 1 further illustrates the management and care of the same admitted patients. Outcome was classified on the Glasgow Outcome Scale (GOS) which was dichotomized as a good outcome (GOS 4-5) or a poor outcome (GOS1-3). A good outcome occurred in 86.1% (GOS 5=457, 21.5%; GOS 4=1374, 64.6%) of patients. A poor outcome occurred in 13.9% of patients (GOS 1= 257, 12.1%; GOS 3= 38, 1.8%). 275 (12.1%) died during the hospital stay. All patients who expired were of severe traumatic brain injury (sTBI) making the mortality of sTBI 60.3%. The condition of most admitted patients improved, with (n=457, 21.5%), making a complete recovery after adequate care, while the condition of 1374 (64.6%) was at least better than it was at admission.

**Figure 2:**
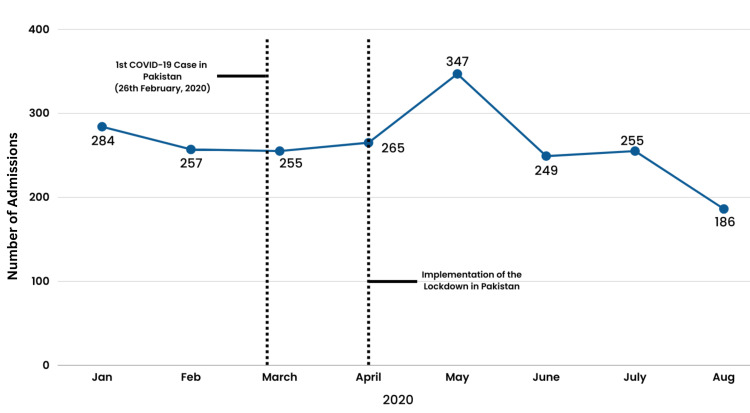
Graph representing the number of patients with traumatic brain injury (TBI) admitted each month, from January to August 2020

Figure 2 illustrates the number of TBI patients who were admitted each month in the study and to determine trends as a result of lockdowns in the volume of head injuries at our department. It was found that while the number of admissions remained stable for the first half of the year up until April 20 (265 cases). When a strict national lockdown was implemented at the end of April 2020, a significant spike in head injury cases in the month of May (347 cases) was observed, followed by a gradual decline in the total number of cases in the months when lockdown restrictions were eased but not ended.

## Discussion

RTAs remain a leading cause of death and non-fatal injuries globally, with over 1.35 million people losing their lives to RTAs every year. Unfortunately, these accidents are most prevalent in low and low-middle-income countries, with over 93% of all fatalities by RTAs occurring in these countries [1]. Pakistan bears a significant burden of such accidents, with legal, social, educational, and administrative lapses making communities at large more vulnerable. Since the outbreak of COVID-19, the world has experienced major disruptions affecting all spheres of life. Pakistan was no exception, with the government implementing multiple restrictions, including suspending international flights and applying screening procedures for passengers, to try and control the spread of the virus. 

The World Health Organization granted COVID-19 the status of a pandemic on the 11th of March 2020, almost two weeks after Pakistan formally registered the first case of COVID-19. On 21st March 2020, all international flights to and from Pakistan were suspended for two weeks, a suspension which was later extended. The COVID-19 screening for domestic passengers was also started on the same day. The first lockdown was imposed in Punjab from 24th March until April 6th. It is important to note that the province of Sindh had already imposed a lockdown at this time, and although initially most COVID-19 cases were reported in Sindh, Punjab quickly took over [12].

This can be explained by a slightly delayed response to the imposition of the lockdown, especially restrictions on public and private transport, as well as a much greater population in the province. The Sindh government restricted movement between 8 AM and 5 PM with the early closure of grocery stores, a move taken much later by the Government of Punjab [13]. Hence, the use of private transport and intra-province movement was much easier in Punjab. Putting this into perspective, we observed no considerable change in the number of hospital admissions for road traffic injuries in a government hospital in Lahore between February and April. This can be explained by the fact that lockdown and other COVID restrictions were not in effect from February till the end of March. 

Interestingly, we observed an increase in hospital admissions in May (even after the lockdown was imposed), before we started observing an expected decrease in hospital admissions throughout June, July, and August. This, of course, coincides with the effects of the first wave of COVID-19 in Pakistan, including the closure of educational institutions including all schools and universities, the closure of restaurants, shopping malls, industry, and most of the service sector. This country-wide shift led to decreased economic output. Moreover, initial lockdown measures were viewed as an encroachment of civil liberties (including practicing religious congregations) and a dampening of financial stability by many [14]. Therefore, the initial response of civil society, business owners, and religious clerics might have contributed to there not being a decrease in road traffic movement and hence reported TBIs in the first few months of COVID-19 remained stable. Consequently, the government intervened by tightening the legislation and ensuring compliance with the lockdown guidelines.

The trend could be expected to be much different in a city such as Karachi, a city in Sindh, where the lockdown implementation measures, especially regarding restricting movement and use of public and private transport were much more drastic, leading to fewer people traveling. According to a study published on analysis of RTA fatalities in Karachi, the number of fatalities reported in March-July 2020 was 35.6% lower when compared to data from the same months for the past year. This could be attributed to the strict restrictions imposed on travel by the Government citing COVID-19. This significantly reduced the traffic burden for inter and intracity transport leading to a reduction in the number of RTAs [15].

Pakistan was not the only country, however, to observe this phenomenon. Studies from Ireland, Greece, and Peru have reported similar trends, with not only a reduction in the number of RTAs but also a reduction in the number of traumatic brain injuries sustained as a result of the RTAs [16,17]. Since TBIs form the overwhelming majority of sustained, and generally more serious injuries (the same study above reported the cause of death in 64.6% of autopsies to be head injury), a decrease in the incidence of TBIs is linked to a decrease in overall fatalities globally [18]. 

A significant segment of the working-class population was forced to work from home or laid off completely. Schools, colleges, and universities were closed, and the tertiary sector including the service and entertainment industry came to a halt. This meant that with little socioeconomic activity, most employed people were not traveling to and from their workplaces, significantly reducing the risk of RTAs. Furthermore, changes in cultural practices and social engagements have also contributed to the reduction of accidents. High-risk community events such as public gatherings, protests, and cultural festivities have been reduced, thus impacting road traffic patterns [19,20]. 

Another finding is the relatively young age of (mean: 28.85) the majority of patients admitted to the hospital for TBIs in our study. These trends are very similar to previous studies reported from Pakistan, other developing countries, and global statistics with RTAs being the leading cause of death in children and young adults aged 5-29 [21-23]. Furthermore, although the overwhelming majority of Pakistan’s population comprises younger individuals, it must be noted that everywhere around the world, the immobilization of the elderly population due to COVID (citing recommended isolation to prevent frequent contact) meant that they were involved in RTAs much less frequently. In fact, our study shows that only less than 8% of hospital admissions for TBIs were for patients 60 or above [24].

Our study also involved traumatic brain injury secondary to non-RTA events including falls, assaults, and other significant events, which could also be impacted (to a much lesser degree) by the COVID-19 pandemic. For instance, the literature shows that the pandemic significantly changed the location of most reported assault-related TBIs from streets and outside to within homes, with an increased incidence of random assaults on middle-aged adults [25]. Other studies showed no change in the incidence of falls at home in the COVID era when compared to the pre-COVID demographics [26,27]. Similarly, the literature has shown violent trauma to have increased significantly in COVID-19 due to a variety of factors [28]. Therefore, while the effect of COVID-19 on all etiologies of TBI cannot be neglected, a reduction in RTAs provides a much more documented and understood reason for the changing trends and demographics of TBIs.

This study has multiple limitations. The data obtained is retrieved from one hospital in Lahore, a metropolitan in Pakistan. Predicting nationwide trends, using data from a single city is extremely difficult. Moreover, since our study looks at the incidence of TBIs in general, predicting a change in RTAs in the COVID-19 era can be a challenge, especially provided that previous studies have shown a change in trends of other causes of TBIs such as assaults in COVID-19 as well. Similarly, since this is a retrospective review of medical records, there is a risk of underestimation of reported cases of RTAs and TBI in general since not all patients sustaining head trauma present to hospitals or report the incident to the police (in case of RTAs). Replicating such studies with a significantly larger sample size and involving multi-center reporting can add weight to the conclusions drawn above. In the post-COVID era, newer studies examine the implications, impact, and possible explanations (including Long-COVID) for shifts in TBI epidemiology worldwide [29,30]. 

## Conclusions

To conclude, the COVID-19 pandemic significantly impacted the demographic trends associated with TBIs and possibly the management of TBIs in Pakistan. These trends can be explained by a reduction in cases of RTAs due to lockdown restrictions, as reported in the literature. It will be interesting to follow if the paradigm shift in the socioeconomic habits of the society as experienced in COVID-19 will persist now that the pandemic is over, and how this will impact the incidence of RTAs and TBI long term. While data from our study provides important demographic details associated with hospital admissions due to TBIs in Pakistan, it must be remembered that these numbers correspond to a tertiary care center from a metropolitan city, and therefore the trends in rural areas (where the majority of Pakistani population lives) can be significantly different as well.
